# Coagulopathy in newborns with hypoxic ischemic encephalopathy (HIE) treated with therapeutic hypothermia: a retrospective case-control study

**DOI:** 10.1186/1471-2431-14-277

**Published:** 2014-11-03

**Authors:** Katie R Forman, Yaser Diab, Edward CC Wong, Stephen Baumgart, Naomi LC Luban, An N Massaro

**Affiliations:** Division of Neonatology, Children’s National Medical Center, 111 Michigan Ave NW, Washington, DC, 20010 USA; Division of Hematology/Oncology, Children’s National Medical Center, 111 Michigan Ave NW, Washington, DC, 20010 USA; Division of Laboratory Medicine, Children’s National Medical Center, 111 Michigan Ave NW, Washington, DC, 20010 USA; Department of Pediatrics, The George Washington University School of Medicine and Health Sciences, 2300 Eye Street, NW, Washington, DC, 20037 USA; Department of Pathology, The George Washington University School of Medicine and Health Sciences, 2300 Eye Street, NW, Washington, DC, 20037 USA; Division of Neonatology, Children’s Hospital at Montefiore, 1825 Eastchester Road, Bronx, NY 10461 USA; Department of Pediatrics, Albert Einstein College of Medicine, 1300 Morris Park Ave, Bronx, NY 10461 USA

## Abstract

**Background:**

Newborns with hypoxic ischemic encephalopathy (HIE) are at risk for coagulopathy due to systemic oxygen deprivation. Additionally, therapeutic hypothermia (TH) slows enzymatic activity of the coagulation cascade, leading to constitutive prolongation of routinely assessed coagulation studies. The level of laboratory abnormality that predicts bleeding is unclear, leading to varying transfusion therapy practices.

**Methods:**

HIE infants treated with TH between 2008–2012 were included in this retrospective study. Initial, minimum (min) and maximum (max) values of International Normalized Ratio (INR), activated partial thromboplastin time (aPTT), fibrinogen (Fib) and platelet (PLT) count (measured twice daily during TH) were collected. Bleeding was defined as clinically significant if associated with 1) decreased hemoglobin (Hb) by 2 g/dL in 24 hours, 2) transfusion of blood products for hemostasis, or 3) involvement of a critical organ system. Laboratory data between the bleeding group (BG) and non-bleeding group (NBG) were compared. Variables that differed significantly between groups were evaluated with Receiver Operating Characteristic Curve (ROC) analyses to determine cut-points to predict bleeding.

**Results:**

Laboratory and bleeding data were collected from a total of 76 HIE infants with a mean (±SD) birthweight of 3.34 ± 0.67 kg and gestational age of 38.6 ± 1.9 wks. BG included 41 infants. Bleeding sites were intracranial (n = 13), gastrointestinal (n = 19), pulmonary (n = 18), hematuria (n = 11) or other (n = 1). There were no differences between BG and NBG in baseline characteristics (p > 0.05). Both groups demonstrated INR and aPTT values beyond the acceptable reference ranges utilized for full tem newborns. BG had higher initial and max INR, initial aPTT, and lower min PLT and min Fib compared to NBG. ROC analyses revealed that platelet count <130 × 10^9^/L, fib level <1.5 g/L, and INR >2 discriminated BG from NBG.

**Conclusions:**

Laboratory evidence of coagulopathy is universal in HIE babies undergoing TH. Transfusion strategies to maintain PLT counts >130 × 10^9^/L, fib level >1.5 g/L, and INR <2 may prevent clinical bleeding in this high risk population.

## Background

Coagulopathy is one of the many consequences of compromised oxygen and blood supply to the neonatal liver and bone marrow after perinatal asphyxia [1–6]. Therapeutic hypothermia (TH), the current standard of care for hypoxic ischemic encephalopathy (HIE) after perinatal asphyxia, is known to slow enzymatic activity involved in the coagulation cascade [7–15]. Although prior studies evaluating the safety and efficacy of TH have not demonstrated increased incidence of major hemorrhage in cooled versus non-cooled infants [16, 17], most studies report high rates of coagulopathy in this patient population, often requiring transfusion therapy [18].

Transfusion therapy and coagulation monitoring during TH is variable between institutions and practitioners. It is unclear what laboratory abnormalities are predictive of bleeding in the setting of hypothermia. Additionally, it is unclear whether transfusion therapy should target normalization of standard tests of coagulation versus a more conservative approach of initiating replacement only after clinical bleeding is observed. Algorithms to optimize transfusion therapy to prevent clinical bleeding while minimizing exposure to unnecessary blood products are needed.

The aim of this study was to identify the thresholds of International Normalized Ratio (INR), activated partial thromboplastin time (aPTT), fibrinogen (Fib) and platelet (PLT) count that are associated with bleeding in HIE infants undergoing TH. Identified thresholds can guide transfusion therapy in this population at high risk for coagulopathy and clinical bleeding.

## Methods

### Study population

This retrospective study was conducted at an outborn level 4 neonatal intensive care unit (NICU) in an academic free-standing children’s hospital. Infants admitted to the Children’s National Medical Center (CNMC) NICU and treated with whole-body TH according to established criteria and methods [16] between 2008–2012 were identified from a departmental database. Exclusion criteria included death during TH (due to incomplete data for evaluation) and concurrent treatment with ECMO (due to exposure to systemic heparinization). This research was performed in accordance with The Declaration of Helsinki and ethical approval was obtained from the CNMC Institutional Review Board (IRB# 00002771). A waiver of informed consent was granted for this retrospective study and all data were collected in compliance with the Health Information Portability and Accountability Act.

### Data collection

Demographic and clinical data were collected from the medical record. Laboratory data including initial, minimum (min) and maximum (max) values of INR, aPTT, Fib level and PLT count (measured twice daily during TH per protocol) were also collected. Samples obtained from heparinized lines were treated with heparinase to neutralize the heparin effect prior to aPTT determination. Infants were stratified into two groups based on the presence or absence of clinically significant bleeding, which was defined a priori (according to the Perinatal Haemostasis Subcommittee of the International Society on Thrombosis and Haemostasis) as any observed bleeding that 1) decreased hemoglobin (Hb) by 2 g/dL in 24 hours; 2) required blood products for hemostasis; or 3) was in a critical organ system (e.g. pulmonary, gastrointestinal or intracranial hemorrhage identified on MRI performed after TH) [19]. Minor bleeding events such as peri-intubation bleeding from an endotracheal tube, coffee ground gastric residuals and minor subdural hemorrhages, likely related to birth trauma, were not included. Information regarding transfusions of fresh frozen plasma (FFP), cryoprecipitate, PLTs, and red blood cells were also recorded. Standard NICU guidelines for transfusion during the study period included maintaining hematocrit levels greater than 35% for infants requiring respiratory support, transfusing PLTs for counts less than 100 × 10^9^/L, and FFP or cryoprecipitate for Fib less than 1.5 g/L and/or overt clinical bleeding. INR/aPTT-based decisions for transfusion therapy remained practitioner dependent, in part stimulating the conduct of this study.

### Availability of supporting data

Readily reproducible materials described in this manuscript, including all relevant raw data, are freely available to any scientist wishing to use them for non-commercial purposes, without breaching participant confidentiality, and may be obtained by contacting the corresponding author.

### Statistical analysis

Descriptive statistics included mean (±95% confidence intervals) or median (range) for parametric and non-parametric continuous variables respectively, and frequencies for categorical data. Laboratory data between the bleeding group (BG) and non-bleeding group (NBG) were compared using Student’s T test, Mann–Whitney U and Chi-Square Tests for independent samples where appropriate. Variables that reached statistical significance (P value <0.05) were further evaluated with Receiver Operating Curve (ROC) analyses in which an area under the curve (AUC) of 1 denotes 100% agreement between predicted and actual outcomes, versus an AUC of 0.5 that demonstrates no discrimination between groups. Cutpoints with optimal specificity and sensitivity were selected from the ROC to identify values that predict clinical bleeding.

## Results

A total of 97 infants treated with TH during the study period were evaluated. Sixteen (16.5%) infants died and 5 (5%) required ECMO during TH and were excluded from analysis. Complete bleeding and laboratory data were collected from 76 infants. The BG was comprised of 41 infants that had bleeding observed from one or more sites including intracranial (n = 13), gastrointestinal (n = 19), pulmonary (n = 18), hematuria (n = 11) or profuse umbilical stump bleeding (n = 1). Bleeding events were significant enough to require intervention by the clinical team; ie ventilator settings were escalated in setting of pulmonary hemorrhage, H_2_ blockers or proton-pump inhibitors were started for gastrointestinal bleeding, and/or coagulation products were given. However, there were no major life-threatening bleeding events requiring surgical or neurosurgical interventions. There were no differences in the baseline or clinical characteristics between the BG and NBG, except that more infants with severe encephalopathy were in the BG (Table 1). Not surprisingly, more infants in the BG group received blood product transfusions and had a greater number of transfusions compared to the NBG group (p < 0.05) (Table 2).

**Figure 1:**
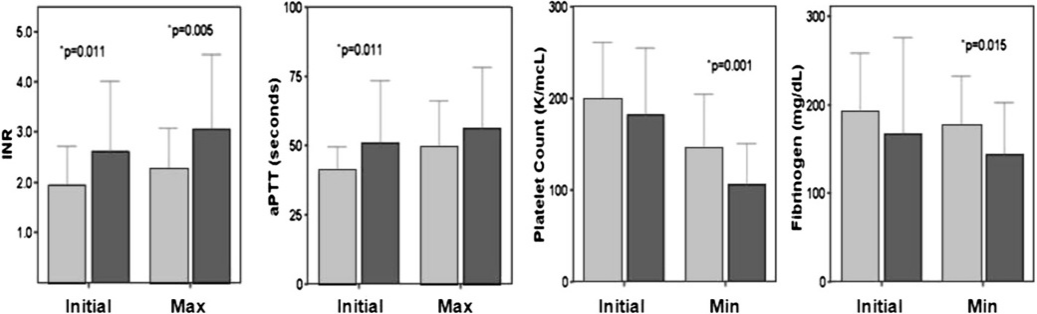
**Each panel demonstrates a bar graph representing the mean ± SD for initial and max/min value of INR, aPTT, platelet count and fibrinogen level as labeled.** The bleeding group is represented by dark bars and nonbleeding group is represented by light bars. The * indicates a p value of significance by Independent Samples T-test.

BG had higher initial and max INR, and initial aPTT than the NBG. Moreover, min PLT count and min Fib level were lower in the BG versus NBG (Figure 1). Other parameters did not differ between groups (p > 0.05).

**Table 1:** **Clinical and demographic characteristics of study population**

	Bleeding	Non-bleeding	P-value
	(n = 41)	(n = 35)	
Birth weight* (grams)	3.376 ± 0.66	3.308 ± 0.69	0.66
Gestational age* (weeks)	38.63 ± 2.11	38.57 ± 1.54	0.884
Gender (n, % male)	25 (61)	22 (62.9)	0.866
Presenting pH	6.95 (6.6–7.34)	6.985 (6.44–7.35)	0.472
5 min Apgar	2 (1–7)	3 (1–7)	0.73
Encephalopathy Grade			0.048
Moderate (n, %)	34 (83)	34 (97)	
Severe (n, %)	7 (17)	1 (3)	
EEG seizure (n, %)	13 (31.7%)	7 (20%)	0.248

Data presented as median (range) except where indicated.

*Mean ± standard of deviation.

**Table 2:** **Transfusion information**

Transfusion type	Bleeding n(%)	Non-bleeding n(%)
Packed red blood cells	15 (36.6)	5 (14.2)
Fresh frozen plasma	22 (53.7)	11 (31.4)
Cryoprecipitate	13 (31.7)	3 (8.5)
Platelets	17 (41.5)	6 (17.1)

Data presented as n(%) of patients in each group who received transfusion with product listed.

**Figure 2:**
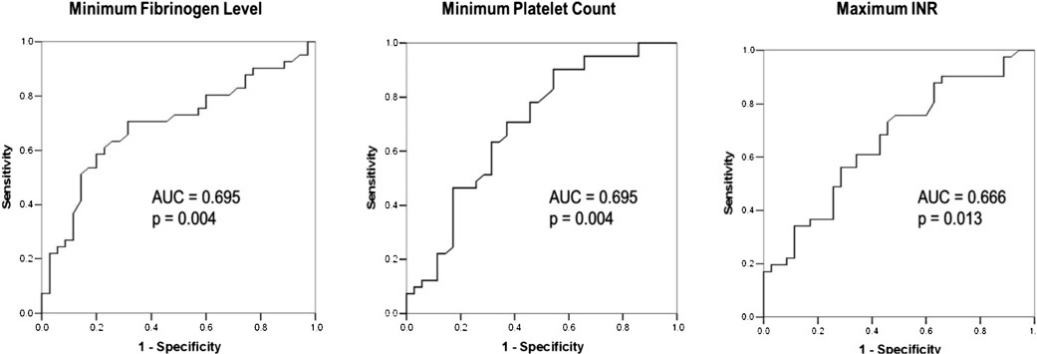
**This figure shows the receiver operator curves used to determine cutpoints for the minimum platelet count, minimum fibrinogen level and maximum INR that predict clinical bleeding.** AUC = Area under the receiver operator curve.

ROC curves are shown in Figure 2. Cutpoints selected from the ROC curves to predict bleeding include min Fib level of 1.54 g/L (71% sensitivity, 68.6% specificity, AUC = 0.695, p = 0.004); min PLT count of 130.5 × 10^9^/L (71% sensitivity, 62.9% specificity, AUC = 0.695, p = 0.004); and max INR of 1.98 (73.2% sensitivity, 54.3% specificity, AUC = 0.666, p = 0.013). The ROC curve for initial aPTT was not significant.

## Discussion

While the safety and efficacy of TH in reducing neurodevelopmental sequelae after asphyxia is well established [16–18, 20, 21], establishing evidence based guidelines for managing coagulopathy during TH can optimize medical care and contribute to better outcomes. While some prior studies have reported platelet counts and aPTT/PT results during hypothermia [7, 22], this is the first study to systematically evaluate the relationship between laboratory and clinical evidence of coagulopathy in newborns undergoing TH. Although major hemorrhages were not observed, which is consistent with prior randomized trials [22] and recent meta-analyses [18], clinically significant bleeding and associated exposure to transfusion therapy were highly prevalent in these critically-ill newborns. The cutpoints provided in this study provide less arbitrary thresholds for transfusion therapy in response to commonly followed tests of coagulation, addressing an important and frequently encountered comorbidity in newborns with HIE.

Coagulation disturbances after birth asphyxia have multifactorial origin. Alterations in blood and oxygen supply to the liver and bone marrow can negatively impact the synthesis of clotting factors and PLTs respectively [1, 3–5]. Disseminated intravascular coagulation (DIC) has also been frequently described following asphyxia in the newborn [2, 6]. Additionally, hypothermia has the potential to impair hemostasis by slowing enzymatic function of the coagulation cascade, impairing thrombin generation, and further triggering DIC [8, 10–14]. Hypothermia also causes PLT dysfunction by decreasing the levels of thromboxane A2 and platelet surface expression of the glycoprotein Ib-IX complex [9, 23]. Bleeding risk, therefore, remains an important problem for infants with HIE undergoing TH. Assessment of bleeding risk is routinely performed by serial monitoring of aPTT, PT/INR, Fib levels and PLT counts. Interpretation of these tests is limited by inadequate data associating these laboratory values with bleeding in the neonatal population. Additionally, aPTT and PT/INR and Fib levels are routinely performed at 37°C in the laboratory, which may not accurately reflect the *in-vivo* condition of a patient undergoing TH. aPTT and PT are likely to be more prolonged at 33.5°C, thus bleeding risk may be underestimated if routine norms are considered in samples that are warmed prior to determination. The unclear relationship between laboratory abnormalities and bleeding risk explains why there is no uniformly accepted protocol for management of coagulopathy in HIE amongst practitioners and institutions. As a significant proportion of the NBG received transfusion therapy, it is presumed that this was done in response to laboratory derangements alone based on the discretion of providers. Whether this practice protected these patients from bleeding risk or whether this represents unnecessary exposure to blood products cannot be answered by the current study.

That the incidence of bleeding reported in this study (54%) exceeds the rates from published trials is likely attributable to our conservative definition of bleeding [19], which was selected to encompass bleeding events felt to be of clinical significance and not confined to only major or life-threatening events. These events either required active intervention and/or had risk for independent sequelae, as in the case of intracranial hemorrhage. Variable rates of intracranial hemorrhage (8–39%) have been reported from the randomized cooling trials [24, 25] and it is possible that risk for hemorrhage can be impacted by variable approaches to correcting coagulopathy. Intracranial hemorrhage, which may or may not be associated with clinical symptoms, is important to recognize and serves as a strong counter-argument to transfusion approaches that only treat overt bleeding. Thus, thresholds for acceptable laboratory derangements are needed to direct transfusion therapy in order to ideally prevent bleeding before it occurs.

While significant associations were found between these routinely used tests of coagulation and clinical bleeding, it should be noted that none of the tests exhibited optimal sensitivity and specificity in isolation to predict bleeding. Thus, a battery of tests must be followed in combination to provide an overall assessment of coagulation status. Investigation into other methods to assess coagulation status in this population is warranted. Thromboelastography, a functional assay of global hemostatic status that can be performed under temperature regulated conditions, shows promise [26] and may improve the approach to assessment and treatment of bleeding risk in this population.

This study has limitations. While the definition of significant bleeding used in this study has been proposed and supported by other investigators, qualifying events by an associated 2 g/dL drop in Hb may be questioned as iatrogenic anemia from frequent laboratory monitoring in the NICU is common. However, no bleeding events were classified as significant in this study based on the association with acute anemia alone (i.e. all required acute intervention and/or were identified in a critical organ system such as intracranial hemorrhage). Thus, we feel that only significant bleeding events were included in these analyses. We were unable to address the temporal relation of laboratory assessments, transfusions and bleeding events in this study. In particular, diagnosis of intracranial hemorrhage was routinely made from MRI performed after rewarming. Thus we used the approach of evaluating bleeding at any point during cooling/rewarming and its association with laboratory values measured over the course of TH. Due to this approach, we excluded infants who died during hypothermia as they would, by definition, have an incomplete and non-comparable observation period. As these infants were likely to have coagulopathy with more severe multi-system disease, their inclusion could have potentially skewed the data and therefore we proceeded with a more conservative approach to evaluate only complete laboratory and bleeding data from survivors. Our transfusion practice during the study period did not call for active transfusion therapy with increased laboratory surveillance when needed to maintain the goals proposed in this study. We have since adjusted our practice based on these data and plan to evaluate whether management of coagulopathy with these proposed transfusion goals reduces clinical bleeding in babies with HIE undergoing TH.

## Conclusions

Clinically significant bleeding is highly prevalent in neonates with HIE undergoing TH. PLT counts <130 × 10^9^/L, Fib level <1.5 g/L and an INR of >2 were associated with increased risk for clinical bleeding in this population. Further study is needed to determine whether transfusion therapy according to these thresholds will reduce the incidence of clinical bleeding in babies with HIE being treated with TH.

## Abbreviations

aPTT: Activated partial thromboplastin time
AUC: Area under the curve
BG: Bleeding group
CNMC: Children’s National Medical Center
ECMO: Extracorporal membrane oxygenation
Fib: Fibrinogen
Hb: Hemoglobin
HIE: Hypoxic ischemic encephalopathy
INR: International normalized ratio
IRB: Institutional review board
min: Minimum
max: Maximum
NICU: Neonatal intensive care unit
NBG: Nonbleeding group
PLT: Platelet
ROC: Receiver operator characteristic
TH: Therapeutic hypothermia.

## References

[1] Bauman ME, Cheung PY, Massicotte MP (2011). Hemostasis and platelet dysfunction in asphyxiated neonates. J Pediatr.

[2] Suzuki S, Morishita S (1998). Hypercoagulability and DIC in high-risk infants. Semin Thromb Hemost.

[3] Shah P, Riphagen S, Beyene J, Perlman M (2004). Multiorgan dysfunction in infants with post-asphyxial hypoxic-ischaemic encephalopathy. Arch Dis Child Fetal Neonatal Ed.

[4] Castle V, Andrew M, Kelton J, Giron D, Johnston M, Carter C (1986). Frequency and mechanism of neonatal thrombocytopenia. J Pediatr.

[5] Roberts IA, Murray NA (2003). Thrombocytopenia in the newborn. Curr Opin Pediatr.

[6] Buchanan GR (1986). Coagulation disorders in the neonate. Pediatr Clin N Am.

[7] Sarkar S, Barks JD, Bhagat I, Donn SM (2009). Effects of therapeutic hypothermia on multiorgan dysfunction in asphyxiated newborns: whole-body cooling versus selective head cooling. J Perinatol.

[8] Michelson AD, Barnard MR, Khuri SF, Rohrer MJ, MacGregor H, Valeri CR (1999). The effects of aspirin and hypothermia on platelet function in vivo. Br J Haematol.

[9] Michelson AD, MacGregor H, Barnard MR, Kestin AS, Rohrer MJ, Valeri CR (1994). Reversible inhibition of human platelet activation by hypothermia in vivo and in vitro. Thromb Haemost.

[10] Reed RL, Johnson TD, Hudson JD, Fischer RP (1992). The disparity between hypothermic coagulopathy and clotting studies. J Trauma.

[11] Reed RL, Bracey AW, Hudson JD, Miller TA, Fischer RP (1990). Hypothermia and blood coagulation: dissociation between enzyme activity and clotting factor levels. Circ Shock.

[12] Rohrer MJ, Natale AM (1992). Effect of hypothermia on the coagulation cascade. Crit Care Med.

[13] Straub A, Breuer M, Wendel HP, Peter K, Dietz K, Ziemer G (2007). Critical temperature ranges of hypothermia-induced platelet activation: possible implications for cooling patients in cardiac surgery. Thromb Haemost.

[14] Wolberg AS, Meng ZH, Monroe DM, Hoffman M (2004). A systematic evaluation of the effect of temperature on coagulation enzyme activity and platelet function. J Trauma.

[15] Mitrophanov AY, Rosendaal FR, Reifman J (2013). Computational analysis of the effects of reduced temperature on thrombin generation: the contributions of hypothermia to coagulopathy. Anesth Analg.

[16] Shankaran S, Laptook AR, Ehrenkranz RA, Tyson JE, McDonald SA, Donovan EF, Fanaroff AA, Poole WK, Wright LL, Higgins RD, Higgins RD, Finer NN, Carlo WA, Duara S, Oh W, Cotten CM, Stvenson DK, Stoll BJ, Lemons JA, Guillet R, Jobe AH, National Insitute of Child Health and Human Development Neonatal Research Network (2005). Whole-body hypothermia for neonates with hypoxic-ischemic encephalopathy. N Engl J Med.

[17] Azzopardi D, Brocklehurst P, Edwards D, Halliday H, Levene M, Thoresen M, Whitelaw A, Group TS (2008). The TOBY Study. Whole body hypothermia for the treatment of perinatal asphyxial encephalopathy: a randomised controlled trial. BMC Pediatr.

[18] Jacobs SE, Berg M, Hunt R, Tarnow-Mordi WO, Inder TE, Davis PG (2013). Cooling for newborns with hypoxic ischaemic encephalopathy. Cochrane Database Syst Rev.

[19] Mitchell LG, Goldenberg NA, Male C, Kenet G, Monagle P, Nowak-Gottl U, Perinatal, Paediatric Haemostasis Subcommittee of the SSCotI (2011). Definition of clinical efficacy and safety outcomes for clinical trials in deep venous thrombosis and pulmonary embolism in children. J Thromb Haemost.

[20] Gluckman PD, Wyatt JS, Azzopardi D, Ballard R, Edwards AD, Ferriero DM, Polin RA, Robertson CM, Thoresen M, Whitelaw A, Gunn AJ (2005). Selective head cooling with mild systemic hypothermia after neonatal encephalopathy: multicentre randomised trial. Lancet.

[21] Shankaran S (2012). Hypoxic-ischemic encephalopathy and novel strategies for neuroprotection. Clin Perinatol.

[22] Shankaran S, Pappas A, Laptook AR, McDonald SA, Ehrenkranz RA, Tyson JE, Walsh M, Goldberg RN, Higgins RD, Das A (2008). Outcomes of safety and effectiveness in a multicenter randomized, controlled trial of whole-body hypothermia for neonatal hypoxic-ischemic encephalopathy. Pediatrics.

[23] Valeri CR, Feingold H, Cassidy G, Ragno G, Khuri S, Altschule MD (1987). Hypothermia-induced reversible platelet dysfunction. Ann Surg.

[24] Shankaran S, Barnes PD, Hintz SR, Laptook AR, Zaterka-Baxter KM, McDonald SA, Ehrenkranz RA, Walsh MC, Tyson JE, Donovan EF, Goldberg RN, Bara R, Das A, Finer NN, Sanchez PJ, Poindexter BB, Van Meurs KP, Carlo WA, Stoll BJ, Duara S, Guillet R, Higgans RD, Eunice Kennedy shriver National Institute of Child Health and Human Development Neonatal Research Network (2012). Brain injury following trial of hypothermia for neonatal hypoxic-ischaemic encephalopathy. Arch Dis Child Fetal Neonatal Ed.

[25] Rutherford M, Ramenghi LA, Edwards AD, Brocklehurst P, Halliday H, Levene M, Strohm B, Thoresen M, Whitelaw A, Azzopardi D (2010). Assessment of brain tissue injury after moderate hypothermia in neonates with hypoxic-ischaemic encephalopathy: a nested substudy of a randomised controlled trial. Lancet Neurol.

[26] Forman KR, Wong E, Gallagher M, McCarter R, Luban NL, Massaro AN (2014). Effect of temperature on thromboelastography (TEG) and implications for clinical use in neonates undergoing therapeutic hypothermia. Pediatr Res.

[CR27] The pre-publication history for this paper can be accessed here:http://www.biomedcentral.com/1471-2431/14/277/prepub

